# Mr. Travers on the Treatment of Diseases of the Eye

**Published:** 1821-09-01

**Authors:** 


*A Synopsis of the Diseases of the Eye, and their Treatment, &c.* By Benjamin Travers, F. R. S. Surgeon to St. Thomas's Hospital. Octavo, pp. 426. London, 1821.

In our last number from page 105 to page 133, we offered a comprehensive, and, at the same time, minute analysis of the *pathological* division of Mr. Travers's excellent work. We now proceed to the completion of our task, by a similar notice of the *therapeutics*, medicinal and operative, which this able surgeon has presented to his brethren, as the fruit of ample experience in ophthalmic surgery. If we have devoted a space, in this Journal, somewhat out of proportion to the size of the work reviewed, it was not without due consideration to the interest of the public—an interest which must always be identical with that of the Journalist himself, where the latter is *guided* by common sense and sound wisdom, rather than *impelled* by the gales of passion and personal feeling. Our reasons then for being minute in our analysis of the work before us are two-fold—first, because ophthalmic science is beginning to spread extensively through all ranks of practitioners; and, secondly, because the expense of the plates cripples the circulation of the volume among those widely scattered members, or humble grades of the profession, for whose benefit *in particular* this Journal was first instituted, and we hope will be ever conducted. Where the *original*, therefore, *cannot* travel, we deem ourselves always bound to provide the best possible *substitute*—where it *can* and *ought* to travel, we hold out the strongest temptation for accelerating its progress.

The third part of the work before us is divided into four chapters, which are subdivided into sections, and occupy altogether about 140 pages of letter-press. In this portion we were much pleased to observe that our intelligent author endeavoured "to seize upon the principles of treatment, to the exclusion of over-minute practical details."

"For it would be idle to suppose that general principles of treatment require to be enforced, after the nature of a disease is clearly pointed out. The maxims and modes of successful practice, so far as they are hitherto known, are accessible to all inquirers of ordinary capacity; and that man is unworthy of his profession who seeks to mystify them, for the purpose of being esteemed wiser than his neighbours." 245.

In the above sentiments we cordially agree with Mr. Travers.

1. *Simple Ophthalmic Inflammation.* Where this is unconnected with constitutional disorder or diathesis, the loss of a few ounces of blood, the use of soothing applications and cathartics, are sufficient to subdue it. Febrile irritation rarely accompanies this form, and when it does, it yields to the same means. In many cases of this kind, general blood-letting is unnecessary. Cupping possesses advantages over leeches, especially when the latter are applied on the eyelids, as they there produce swelling and discolouration—or even troublesome erysipelatous inflammation. In urgent cases, however, the lancet must be used, in order "to make the system sustain and feel a reduction of power." Bleeding from the angular vein and scarification of the conjunctiva are highly beneficial in chronic, but objectionable (especially the latter) in acute inflammation. In respect to lotions, he does not consider *medicated* ones of any material efficacy. Cold applications, though they are momentarily agreeable to an acutely inflamed organ, are nevertheless followed by reaction, with an increase of heat and pain.

"When, however, the acuteness of inflammation has subsided, and the sensibility of the part is in proportion diminished, the effect of cold is only tonic, and has a salutary tendency to restore the balance of circulation, I therefore decidedly prefer, as a general practice, a tepid application in the painfully acute stage of inflammation, and I appeal to general observation in proof of its efficacy in promoting a grateful sense of coolness, and a more permanent relief from pain." 250.

Our author has found narcotic lotions and fomentations very various in their effects on different people—irritating in some, anodyne in others. Though warm fomentations are best in the acute stage, they should not be continued longer than is necessary, otherwise they prove injurious. When the vascular congestion and morbid sensibility are reduced, cold lotions, of a slightly tonic quality, may be substituted for the warm, with advantage. The sulphates of alum and zinc are the best. The œdematous elevation of the conjunctiva indicates feeble action, and is often combined with gastric and hepatic derangement. "Solution of tartrite of antimony given at short intervals operates very beneficially in reducing it."

Those ophthalmiæ which assume a chronic atonic character, ab origine, being partial in extent, void of pain, and almost solely congestive, are often restored to sanity by a single stimulus, as the vinum opii, or, which Mr. Travers considers to be equally good, a drop or two of zinc, or lunar caustic solution, or water impregnated with calomel. "Some old women use their urine with admirable effect in these cases."

In certain habits, ophthalmia, whether local or constitutional, becomes worse under the usual depletory measures, the irritability increasing as the strength fails. Here it is that combinations of calomel, opium, and antimony or ipecacuan, are peculiarly serviceable.

"In the treatment of simple acute ophthalmia the object to be kept in view is the soundness of the cornea; the organ is in no danger of deeper injury. The main indication for an activity of treatment beyond that successfully adopted in ordinary cases, is furnished by the state of this membrane. Where the sclerotic conjunctiva is much raised, and the surface of the cornea has in any degree lost its polish, and still more when lymph is effused in or upon the cornea so as to obscure vision, the anti-inflammatory measures must be as vigorous and decided as the integrity of the organ is important. Blood-letting and blisters, calomel, antimony, and the neutral salts comprise all the requisite means." 254.

2. *Strumous Ophthalmia* is almost always atonic and constitutional. Speaking generally, blood-letting is unnecessary, rough and depressing purgatives, injurious. Warm applications are useless—blisters to the nape of the neck and behind the ears very beneficial, especially where the corneal surface is affected, and the consequent sensibility augmented. Issues and setons are also of great avail.

"If the cornea be opaque, calomel, or the blue pill, or the oxymuriate of mercury should be exhibited in combination with opium, slightly to affect the system. The efficacy of the mercurial mainly depends on its combination with opium; it irritates too much if administered alone in quantity sufficient for the purpose." 260.

Here our author introduces a synoptical sketch of the treatment in each particular form of the strumous ophthalmia, which the surgeon will consult with advantage. In convalescence after these forms of ophthalmia, the patient should use some of the following medicines:—infusion of roses—cascarilla—calumba—cinchona—steel—rhubarb and soda—with tonic collyria and gently stimulating ointments—nutritive diet, country air, shower or sea bath, in summer.

3. *Acute suppurative Inflammation of the Conjunctiva.* This is the most dangerous form; its sequelæ being lingering, and sometimes difficult of removal. It is either mild or vehement. In the mild, the cornea is not endangered, unless the disease be exasperated by stimulants. "A very slight haze of the cornea is the worst direct result of it." There is not that excessive swell of the lids, that intense pain, nor that profuse secretion, which characterizes the vehement acute form of the disease. Nevertheless, these symptoms exist in a degree requiring immediate and active treatment. The alum solution should be early substituted for the emollient fomentations necessary in the acute period. The solution should be directed in a gentle stream over the conjunctival surface, from a syringe furnished with an ivory pipe introduced at the temporal angle of the lids without forcibly separating them. Simple purging and abstinence are generally sufficient to allay the febrile irritation, which is moderate. Topical bleedings and a suppurating surface, opened by blistering the back of the neck, are of great efficacy. Pain and irritability having subsided, the discharge becomes gleety, and the conjunctiva flaccid, tonics, especially bark and acids, are useful. While the cornea appears clear and bright, there is no cause for apprehension.

"The vehement acute suppurative inflammation is sudden in its attack, accompanied with most severe darting pains; the upper lid is in a few hours prolonged upon the cheek, owing to the infiltration and enormous swelling of the tissue connecting the conjunctiva to the tarsus. The cornea is nearly concealed by the fold of conjunctiva which overlaps it all around, and the corneal surface is dusky. The system sympathises, chilliness is succeeded by a hot and dry skin, and the pulse is frequent and hard. The instant relief of a large venesection is indescribable. The pain is mitigated, if not removed; the pulse softened, and the patient sinks into a sound sleep, and perspires freely. Upon inspection we observe the high scarlet hue and bulk of the chemosis sensibly reduced, and the cornea has a brighter aspect." 265.

A single blow will rarely annihilate this disease, especially if it arise from contagious matter. Repeated bleedings, brisk cathartics, nauseating doses of emetic tartar, will often be indispensable.

"The discharge, at first ropy, viscid, and sparing in quantity, becomes thin, gleety, and more abundant; as the swollen lid subsides, the conjunctiva sinks and becomes pale and flabby; and if, at this period, the pain and febrile irritation being past, the cornea retains its tone and brightness, all is well; the disease has given way, and a careful but prompt exhibition of tonics, with the use of cooling astringent lotions, will prevent its lapsing into a chronic form. But if, when the lowering practice has been pushed to the extent of arresting acute inflammation, the patient being at the same time sunk and exhausted, the cornea shews a lack-lustre and raggedness of its whole surface, as if shrunk by immersion in an acid, or a grey patch in the centre, or a line encircling or half encircling its base, assuming a similar appearance, the portion so marked out will infallibly be detached by a rapid slough, unless, by a successful rally of the patient's powers, we can set up the adhesive action so as to preserve in sitû that which may remain transparent. 267.

It requires great judgment to know how far and no farther we are to proceed with depletion in these cases. Our author does not agree with those who have taken sixty or seventy ounces of blood at one time, from a patient. Perhaps, however, Mr. Travers does not make allowance for the class of patients upon whom these large detractions have been practised. It is not impossible that the surgeons alluded to may have been as capable of appreciating Falstaff's adage respecting valor and discretion, as even Mr. Travers himself. A man should state the result of his own experience, but be cautious how he censures the practice of others; especially if situated in widely different circumstances. It is but justice to say, that Mr. Travers very rarely makes any allusions to mal-practice in others, and then in a very delicate manner.

4. *Secondary Diseases of the Conjunctiva.* The granular state of the tarsal conjunctiva is a very common result of mild suppurative ophthalmia. Here the lid should be everted, and the projecting granules shaved off from the surface and orbitar edges of the tarsus, with a keen edged lancet, or, if peduncular and prominent, they will be more conveniently snipped off with the flat scissars, taking care to avoid injuring the continuous membrane. When there is an additional vascularity of the cornea, with consequent opacity of the conjunctival covering, "a section of the membrane should be made at one line's distance from the margin of the cornea." In aggravated cases the operation requires to be repeated.

"After the excision of the granulations and the division of the conjunctiva, a solution of the sulphate of copper, or some astringent, is very advantageously employed in the way of injection. A few drops of the liq. plumb. sub-acetatis, or the tinct. opii vinos, are often highly effective." 272.

The lunar caustic is often useful in preventing the regeneration of the granulations. In cases of fungous protrusions or flap-like elongations of the conjunctiva, the treatment consists simply in the excision of the tumours, which is most conveniently done with a lancet-shaped knife, cutting on both sides. The same treatment is applicable to pannus, the elongated valvula semilunaris, and the caruncular excrescences which sometimes form in clusters between the tarsus and globe.

5. *Pterygium.* The fleshy pterygium is sometimes a chronic or stationary disease, producing little inconvenience, and not interfering with vision. In such cases Mr. Travers advises its being let alone.

"When, by its progress, it is encroaching upon the sight, it should be raised by dissection as close as possible to the margin of the cornea, and the relaxed portion of the membrane removed by an incision mid-way between the base of the pterygium and the cornea, and concentric to that membrane." 274.

In this operation Mr. Travers prefers the cornea knife to the scissars. It is inadmissible to meddle with any portion of pterygium that may have encroached upon the cornea. The tendency to reproduction may be checked by the application of the caustic pencil to the section of the tumour.

"The treatment of the membranous pterygium consists in nipping up a crescentic portion of the opaque membrane as near as convenient to the cornea, and freely excising it with a pair of curved scissars. The extremities of the line of excision both in this and the former species should extend beyond the diseased part." 275.

*Diseases of the Cornea.* The lamellæ of horny substance in the cornea have no vessels proper to themselves, but derive them from the covering and connecting cellular tissues. It is rare to see red vessels in the interlamellar texture of the cornea; but deposits of adhesive matter and pus are frequent. The cornea is also rendered turbid by congestion in the vessels of its covering or connecting texture—and thus may be said to be inflamed. The cornea, presenting an onyx of adhesive matter, and being thus rendered opaque, indicates strongly the practice for reducing inflammation.

6. The *superficial ulcer* is commonly attended with much inflammation of the conjunctiva, and, by continuance, of the sclerotica. The pain is often spasmodic, and relieved by profuse lacrymation at intervals. Opium should be so combined as to operate on the skin, and the bowels must be kept freely open. Touching the ulcer with solution of arg. nit. is the best local treatment. Warm fomentations afford temporary relief. "It will be found advantageous, if not indispensable, to prevent relapse, to affect the system with mercury, where the inflammation of the sclerotica is intense."

"The indolent and the deep sloughing ulcer may be touched once or oftener with the caustic pencil, or washed once a day, or oftener, with the solution. The cleansing of the ulcer and the opaque adhesive circle is the sign for a less frequent use of it, and the deposition of new matter, undergoing a vascular organization, renders its further use hazardous. The occasional use of leeches is often a necessary accompaniment to this treatment. The administration of tonics and sedatives is at the same time essential." 279.

7. When the *hypopion* rises towards the pupil, and the ulceration of the cornea is extending, Mr. Travers advises its discharge by a section near its margin. The *procidentia iridis* from ulcer, if small, should be touched with a fine point of caustic—if large and extending, it may be snipped off with a pair of curved scissars, and the caustic pencil applied. The chronic interstitial ulcer requires only stimulant and astringent injections, with blisters, bark, opium, good air, and attention to the chylopoetics.

8. Upon *strumous nebula of the cornea*, Mr. Travers makes some excellent observations, especially respecting the employment of mercury in this and other affections of the eye. No form of recent opacity is so intractable as this. The hydrarargyrum cum creta, or the oxymuriate, in small doses, will sometimes succeed better than other forms, and the combination of calomel with antimony better than with opium. Mercurial frictions, in constitutions which evince an insusceptibility to mercury internally, should be employed; and when once it is decided that the mercurial action should be set up, nothing but the clearest demonstration of the patient's inability to support it, should interfere with the full and fair execution of the plan.

"A character notoriously abused by indiscriminate excess, is in much danger of being further injured by half measures. This I think has been the case of mercury. It is not the most delicate frame which is most ready to admit or least able to support it; and it is not the quantity consumed, but the quantity absorbed, which is to be taken into account by the practitioner. The progress of disease during its exhibition is no argument against its continued employment; in this view, unless the system be fairly under its influence, all that has been given goes for nothing; nay, I have had occasion to see many cases in which, after all the signs of absorption were manifest, its operation upon the disease was for a time unobserved, or was null, and was yet ultimately all that could be wished." 284.

On the other hand, an alarming degree of arterial excitement, or certain morbid appearances of the organ, not looked for in the natural and ordinary course of the disease, would determine the practitioner to suspend the medicine.

9. *Staphyloma*, if purely corneal, producing deformity or irritation and inflammation, should be excised. "The ligature passed through and including two-thirds of the diseased cornea, by means of a curved needle, assists the operation by steadying the globe. If the staphyloma be from dilatation, the iris will be left—if from breach, it is compacted, and removed with the cornea.

10. In the *conical cornea*, the discharge of the aqueous humour is useless, and so are all applications to arrest the disease. Mr. Travers has found repeated blisters, and the more powerful tonics, as steel or arsenic, decidedly serviceable. To these may be added cold bathing, and the practice of opening the eyes in cold spring water. The tubular spectacle frame with a pupillar aperture affords more aid in correcting the vision, than any form of lens.

11. *Sclerotitis*. Our readers will remember that the symptomatology of the diseases, now under consideration, has been laid down in our preceding number, and that number should be constantly referred to, while consulting this article, which confines itself to therapeutics. Sclerotitis is rarely a primary disease. It is by no means so decidedly influenced by mercury as the iritis. The subjects of it are usually reduced and irritable in a high degree, from suffering with rheumatic inflammation in the elbow, knee, or ancle-joint.

" Though it is necessary to use mercury with more reserve than in other forms of inflammation, to suspend its operation at invervals, and allow the system to recover from its immediate effects, yet its exhibition will be found, in the majority of cases, indispensable. The rude and profuse employment of it hurries on the disease, and the extension of the inflammation to the interior tunics ultimately destroys the organ. The nitric acid may often be exhibited with marked benefit, in the intervals of the mercurial action." 289.

The oxymur. hydr. in doses of an eighth to a twelfth of a grain, or five grains of the hyd. c. creta, are most valuable and beneficial in these cases. The Dover's powder, conium, henbane, and extract of sarsaparilla, either dissolved in decoction, or taken freely in the solid form, are useful auxiliaries.

On the subject of choroid and iritic inflammation, Mr. Travers' sentiments are well known, both through the medium of his own works, and the former series of this Journal.

"One full blood-letting or more should be premised in the acute stage of the disease; and topical blood-lettings during the exhibition of it, are generally required at short intervals. I have now and then found that the incipient inflammation, where it has extended from the conjunctiva, yields to a copious venesection and two or three brisk doses of calomel and rhubarb, followed up by the infusion of senna; but, generally speaking, the system must be made to feel the influence of mercury before the disease is permanently subdued. The inflammation which has proceeded to the effusion of adhesive matter, never, in my experience, yields either to the lancet, continued nausea, or full purging; and it is remarkable that the cases presenting this termination of inflammation are always most sensibly and immediately benefited by the remedy in question, whether the cornea or the iris be affected, or any other texture of the body." 291.

After some remarks on the action of mercury in different constitutions, our author closes this section with the following important observations.

"But if any two facts are well established in modern medicine, I apprehend they are these:—first, the power of mercury to arrest acute membranous inflammation, both prior to, and after the effusion of adhesive matter; and secondly, its power rapidly to remove, by an excitement of the absorbing system peculiar to itself, the newly effused adhesive matter. If these facts are admitted, then the propriety of its use is indicated in iritis, as in carditis, pleuritis, peritonitis, and the only practical question that can arise respecting it is how far the patient's strength is equal to support the remedy. There are, I admit, states of the organ as well as of the constitution in which it cannot be borne, and no sooner is its influence felt, than the inflammation threatens disorganization, and if the plan is persevered in, quickly runs on to it. The globe becomes enlarged or misshapen, the sclerotica assumes a livid hue, and the veins a state of varicose congestion; sometimes the eyeball suppurates, and the little remaining vision is completely extinguished. In cases where age, or the existence of other diseases, or the already excessive use of mercury, has greatly enfeebled the powers of the system, it must be used, if ventured upon at all, very sparingly, or with intermissions, and the system must be supported by every admissible means, both of nourishment and medicine, during its employment." 292.

12. *Amaurosis.* This comprehends both functional and organic derangement of the *sentient* system of the eye, impeding vision. The symptomatology and pathology of amaurosis will be found in page 116 *et seq.* of our last number. The functional amaurosis is a very diversified class of disease, and being the effect of some morbid action in the system at large, or some important organ, these last are the proper objects of medical treatment. Thus amaurosis from gastric disorders, plethora, irritation, &c. are remediable; whereas, that from paralysis, the sequel of fever or epilepsy, or severe constitutional disease, is a hopeless form of the malady.

The proper functional amaurosis, if treated at a very early period, is very often cured. The extreme of light or temperature, and the over-exertion of the organ are the chief causes, and the removal of these is obviously necessary to the cure.

"The removal of an irritating or oppressing cause will often effect a sudden and marked relief, as by clearing the intestinal canal of vitiated secretions therein accumulated, by restoring the digestive functions labouring under manifest derangement, or by taking away blood where the necessity is indicated. I have seen an incipient amaurosis distinctly arrested by the extraction of a diseased tooth, when the delay of a similar operation had occasioned gutta serena on the opposite side two years before." 299.

The treatment of amaurosis is almost exclusively constitutional; and our author wisely attaches no credit to the farrago of external remedies, as stimulant vapours, drops, ointments, and etherial embrocations. Cupping and setons will be occasionally necessary, for very obvious reasons; and blisters will be found useful in almost every case. This last remedy should be frequently repeated, and alternately applied over the superciliary ridge, upon the temple, mastoid process, or nape of the neck, as may be judged most necessary. There is great difference of susceptibility under the action of blisters. Mr. Travers has known the irritation and discharge of an efficient one, not bigger than a crown, sink the powers of a delicate female for days, especially where the retina has been weak. Such cases are yet more affected by the direct loss of blood, even in the smallest quantity. Our author has not seen a single instance of benefit from electricity in this disease.

Mr. Travers very justly remarks that a strong and delu sive similarity often prevails between the signs of diseases, resulting from conditions diametrically opposite. Thus the treatment in cases of general plethora and cerebral compression will be evidently depletive; whereas, in cases of undue determination of blood to the organ, very common after deep-seated chronic inflammation, or distress from over excitement, the vessels have lost their tone—an effect decidedly increased by depletion. Our author has never seen any benefit derived from antispasmodics, nor from the emetic practice, recommended by high authority.

"The cases of gastric disorder to which it is especially applicable are most benefited by a long continued course of the blue pill, with gentle saline purgative and tonic bitters." 304.

After the visceral functions are regulated, by blue pill, colocynth, rhubarb, or aloes, we are to exhibit general tonics, as the mineral acids, bark, steel, (when admissible,) and arsenic.

"When the amaurosis is recent and sudden, and either the signs of an obscure inflammation are present, or only the amplitude and inactivity of the pupil correspond to the patient's history, the indication is less simple; mercury should be introduced with all convenient rapidity into the system, I mean so as to ruffle it in the least possible degree. No advantage is obtained by salivation, on the contrary I think it hurtful; when mercury is beneficial its efficacy is perceived as soon as the mouth is sore. I have seen it tried, and have myself tried it in many cases of perfect amaurosis without the smallest advantage; but in cases of recent occurrence, imperfect, but rapidly progressive from bad to worse, I have been witness to its power in suddenly arresting the disease in too many instances, not to entertain a far higher opinion of it than of any other article of the materia medica." 305.

Entire repose of the organ is necessary, with the natural tonic, viz. a pure, dry air, the cold bath, horse exercise, nutritious diet, early and sufficient rest, agreeable society. "These are of greater avail than drugs."

The 4th section of this chapter is on diseases of the eyeball. The treatment of injuries from external violence, falls under the head of inflammation, and need not be alluded to here. Neither shall we touch on the operation of extirpating the eye-ball, but confine ourselves principally to what may be considered *medical surgery*, as all those who undertake the higher operations on such a delicate and important organ as the eye, will naturally possess themselves of the original work. The same reasons will induce us to pass over our author's section on artificial pupil entirely, and to touch on the extensive class of operations for cataract very lightly.

Mr. Travers remarks that it has been customary for oculists, where one eye has been affected with cataract, to advise the postponement of the operation till the other eye becomes dark. This he combats, because the cataractous eye is strongly disposed to become amaurotic on the supervention of accidental inflammation, besides the danger of the retina losing its vigour by the permanent exclusion of light.

"It would be incorrect to say that the operation was unadvisable in all cases of cataract in which the patient has no sense of light, for it is possible that the density of the lens may be such as absolutely to exclude the light, and that the motions of the iris may be therefore suspended, or from some degree of pressure of the lens, or adhesion of the uvea to the capsule, that the pupil may be undilated, and the circumference of the lens permanently covered. But undoubtedly a case of this description is unpromising. A strong sense of light by which at least to know the direction in which it enters the apartment—to be sensible of its falling on the eye, and of a shade, as the hand for example, intercepting it, with a corresponding freedom of motion in the pupil, is the most favourable state for the operation. There is in this case perception enough to determine the sensibility of the retina, and not enough to occasion the unsteadiness of the globe. If a patient has vision, the eye is irritable to light, and involuntarily rolls as far as possible towards the nose on the introduction of the instrument, one of the greatest perplexities in the operation." 315.

Of couching, extraction, and absorption, the first and most ancient, is now seldom practised. It is only applicable to cataracts of firm consistence, and where there are serious impediments to the more eligible mode of extraction.

"The couching needle may be passed through the sclerotica at a line's breadth from the cornea, and a little below the horizontal diameter, so as to avoid the long ciliary artery; or through the inferior part of the cornea and pupil; and the lens may be depressed vertically or horizontally. The term 'reclination' has been applied to the latter method. In both cases the lens must be hitched into a breach of the vitreous humor below the border of the pupil. Its anterior capsule, and the capsule of the vitreous humor, must be divided or torn through, to render the operation effective. The lens corresponds in diameter to the iris, and there is therefore no natural space into which it can be depressed. The posterior capsule, identical with that of the vitreous humor, must be lacerated to admit of its dislocation backwards and downwards; and if its anterior capsule was left entire, it would become a secondary capsular cataract, and require a subsequent operation." 317.

The operation of couching through the cornea (keratonyxis) possesses, in our author's mind, no advantages over that through the sclerotica, the injurious consequences attending the perforation of the latter and choroid, being either imaginary or resulting from unskilful management.

"The real objection to couching is the ultimate step of the operation, viz. the breaking up of the fine texture that fills the globe by the forcible depression of the lens. Whether it be depressed edgeways or breadthways, makes no difference in the result; it must still occupy a breach in the cells of the vitreous humour, and must derange and disorder that delicate texture and those connected with it. A slow insidious inflammation marked by a gradual development of the symptoms of disorganization, viz. congestion of vessels, turbid humors, flaccid tunics, and palsied iris, is too often the consequence. The sight, instead of improving when the immediate effects of the injury are passed away, remains habitually weak and dim, or declines and fades altogether." 319.

Absorption is only to be attempted where the cataract is flocculent, the perfection of which operation consists in making the free central aperture by laceration of the anterior capsule the preliminary operation. The more minutely the lens is broken up and divided in its texture, and the more its fragments are dissipated in the anterior chamber, the quicker the progress of absorption; and the softer the texture of the lens, the more readily and safely is this object accomplished.

"I pass over the description of an operation which consists in the introduction of a knife, whether through the cornea or sclerotica, for the purpose of cutting up the hard crystalline *in sitû*, and throwing the slices into the anterior chamber; and I mention it only by way of caution, if caution be necessary, against a measure so desperate and ill advised." 320.

It is especially to the cataract of infancy, which is fluid or flocculent, that the operation of absorption is applicable. Here indeed there is no alternative, for its consistence does not admit of depression, and the eye is too unsteady to admit of extraction with safety.

"It is impossible to conceive a more simple, sufficient, or gratifying operation than that of Mr. Saunders, if the intention is perfectly executed. I have now enjoyed extensive opportunities of ascertaining its value; having operated, during a period of ten years, upon children of all ages from four months upwards, and I do not hesitate to affirm that it ranks in my estimation as one of the finest discoveries of modern science." 321.

But the operation of extraction is by far the most perfect ever devised for the cure of cataract. It is, however, difficult; the several modifications suggested at different times, owing their origin to the disappointments and defeats which operators have met with in learning to execute it with success. The Baron de Wenzel confessed that he "spoiled a hatfull of eyes," before he had learnt to extract. The main impediment to the success of the operation, Mr. Travers thinks, is—"a section of insufficient magnitude." The easy extraction of a cataract, like the easy extraction of a stone, almost invariably does well, and the difficult and forcible removal of either as certainly augurs unfavourably. The enlargement of the section, if too short, is difficult, and always dangerous to the iris in the collapsed state of the cornea. It is attended, moreover, with imminent risk of a laceration, from the want of due support, of the vitreous capsule, the loss of a portion of this humour, and the consequent sinking of the lens behind the iris. The free escape of the vitreous humour, from imperfect section, may embarrass the operator, and induce him to leave the cataract, in the hope of its absorption, or of removing it at a future time—an erroneous practice in Mr. Travers' opinion. "For as soon as the wound closes, the cataract is raised by the renewal of the aqueous humour, and pressed forward upon the iris." Our author has seen inflammation speedily supervene, in such cases, terminating in suppuration and destruction of the eye. It is a point of importance, our author thinks, that the section should not be carried so low as to verge upon the sclerotica, and thus leave the corneal margin of an insufficient breadth for union. Mr. Travers prefers the section midway between the pupil and margin of the cornea.

Soft, semi-transparent, and unadhering capsular cataracts may all be conveniently extracted, as they pass through a smaller section, and the capsule is easily laid hold of with a hook or forceps.

In respect to instruments, every man must decide for himself. The knife of Professor Beer of Vienna is that which Mr. T. is in the habit of using. The preparative and after-treatment is of great consequence, in the issue of the operation. Purgatives and abstemious diet should precede; and if there be fulness of the vessels of the head, the patient may be cupped the day before operation. "It is a matter of some importance to examine the section, and adjust it accurately before finally closing the eye." A slight friction of the lids assists the pupil to recover its figure, and dissipates any small floating particle of lens. The sitting posture, in an easy chair, is most favourable after the operation, until the patient feels fatigued, and desires to go to bed. Confinement to the latter produces great restlessness.

If the patient complains of pain on the evening of the day of operation, a full blood-letting removes it, and should not be omitted. Mr. T. never gives any opiates. A light bandage passed round the night-cap is sufficient covering for the eyes. Compresses are better omitted. For the inflammation that may succeed the operation, topical blood-letting and blisters are sometimes necessary; but a strict antiphlogistic regimen is always proper. "There is often an irritability to light, an aversion to open the eye, which is removed by two or three brisk doses of calomel."

"The inflammation of the iris, the interstitial ulceration and opacity of the cornea, the separation of the edges of the section by the intervention of another texture, the redundant deposit of lymph in the section, or the ulceration of its edges, are the mischiefs which occur after unfavorable extractions. Blood shed by a wound of the iris in the anterior chamber is quickly absorbed. Where it has even filled the entire chamber, I have found the aqueous humor clear on the succeeding day.

"The coalition of the iris and cornea adjoining the section is the result of a prolapsus or a lesion of the iris. The iritis may be vehement and proceed to amaurosis, or it may terminate favorably under the action of mercury, in constricted pupil. The dimness of the cornea, if any, is slight and transient, except an interstitial herpetic ulcerative action, connected with a bad condition of the edges of the section be present, when the cornea takes on an opacity of a very intractable kind. The sclerotica is in this case inflamed, and very minute depressions appear on the surface of the cornea, which undergoes a total loss of brilliancy, although it remains obscurely transparent. The restoration of smoothness to the surface does not diminish the lacklustre appearance of the membrane. The patient has a perception of light, but no vision of objects. In fact, the cornea precisely resembles that of the dead subject. Mercury is of uncertain efficacy in this case, which fortunately is very rare. Time and tonics do most for it." 331.

We shall pass over the important chapter on the various operations for artificial pupil, because we cannot give a satisfactory analysis of it, and because the work will be in the hands of all surgeons who practise such delicate operations on the eye. We shall therefore, in the remainder of this article, which must soon come to a close, advert only to minor points of opthalmic surgery, as being more generally attended to by practitioners of all ranks. The last chapter is on diseases of the appendages of the eye.

*Hordeolum.* This should be discharged with the point of a lancet, and poulticed or bathed with a slightly astringent wash. The redness of the tarsi, thickenings of the conjunctiva, and small cuticular denudations that result from frequent formations of stye should be treated with dilute nitrated ointment of mercury, and alum or zinc washes.

*Lippitudo.* The acute form generally yields to a single stimulant application; but the chronic lippitudo is a very deforming disease, and often intractable.

"The vessels of the palpebral conjunctiva are turgid, and at length varicose, the membrane a little overlaps the thickened tarsal border; this is partially if not quite denuded of cilia, and small surfaces of the adjoining cutis are excoriated. The follicles are plugged, and here and there is one so much distended by inspissated mucus, as to occasion acute inflammation. These should be opened with the point of the lancet, and the white consolidated secretion removed, the conjunctiva should be occasionally scarified, and the meibomian borders stimulated by one of the ointments above-named. The tarsal edges should also be frequently bathed with an astringent lotion. In the aggravated and obstinate cases of lippitudo, where the conjunctiva is altered in its texture, the sulphate of copper lightly carried over the thickened conjunctiva and ulcerated border of the tarsus, is highly useful; and stimulant solutions of copper, zinc, lunar caustic, or sublimate, applied by a camel-hair brush to the tarsal edges before smearing them with the ointment, are likewise advantageous." 352.

At the risk of being accused of heterodoxy, Mr. Travers asserts that the "golden ointment" is an excellent remedy, and "that the inventor of this arcanum deserves well of his country." The great evil, of course, consists in the *abuse* of quack, as of other medicines.

*Inchiasis.* When the cilia are inverted from a diseased growth, they must be kept plucked until, by the improved condition of the hair gland, the disease is removed.

*Entropion.* The inverted eyelid has been treated successfully on the plan recommended by Scarpa, in nine cases out of ten—that is, by the removal of a fold of skin with a pair of scissars from the surface of the eyelid.

*Ectropeon.* The ordinary ectropeon is cured by the excision of a portion of the thickened or redundant conjunctiva which occasions it.

*Obstruction of the Lacrymal Passages.* Notwithstanding all that has been written by eminent surgeons, the practice, in this class of diseases, is unsettled and unsatisfactory.

"In proof of this remark, I may observe that nearly all the schemes hitherto suggested have been executed within my knowledge by different surgeons, viz. the small probe and injecting syringe of Anel, the sound and syringe for the nasal duct, the seton of silk or catgut, the bougie or nail-headed style, the metallic tube, &c. In Paris, M. Dubois employs the silk seton of Mejan, M. Dupuytren the permanent tube of Wathen, M. Roux the mesh seton introduced by means of a watch-spring from the sac. M. Beer, of Vienna, uses, for a seton, a coil of catgut, such as is used for fiddle-strings, Among the surgeons of this town, Mr. Ware's style is chiefly in use, although the practice is evidently losing credit." 359.

Mr. Travers thinks that the effect of a severe cold in the head, producing a coryza and a troublesome watering of the eye, may enable us to form a pretty accurate idea of the cause of a permanent stillicidium. A state of vascular congestion and intumescence, if long continued, may lead to permanent thickening of the membrane lining the duct, and thus occasion a stricture of its calibre. An adhesive process, whether primary or consecutive to the states of suppuration and ulceration, finally closes the duct, and renders it impervious. "The obliteration of the canal by a degeneration of the membrane into a texture resembling cartilage, is a secondary morbid change, and only the result of long continued obstruction." Abscess, though often the consequenee of obstruction in the duct, may take place totally independent of obstruction, though causing a temporary one. A free opening of the sac, for the purpose of discharging its contents, shortens the sufferings of the patient, and saves the skin; but unless previous symptoms demonstrate stricture, the abscess does not warrant the employment of any farther measures.

Mr. Travers's practice, in cases of lacrymal obstruction, is simply the passing a moderate sized probe into the nose, after the incision of the sac, and then reducing the inflammation of the parts.

"A set of silver probes, of about five inches long, varying in size, flattened at one end, and slightly bulbous at the point, are the instruments I use for the purpose of restoring the passage. The probe is introduced with perfect facility by one who is familiarly acquainted with the anatomy of the part, from either of the puncta lacrymalia, into the corresponding nostril, where no obstruction is offered to its passage. If the punctum be constricted, it is readily entered and dilated by a common pin; and upon withdrawing it, by one of the smaller probes: the direction and relative situation of the lacrymal ducts, the sac, and the nasal canal, point out the proper course of the instrument. It is confirmed by its advance without the employment of force, and the sensation conveyed by the free and unencumbered motion of its point; until the point is fairly within the sac, it is necessary to keep the eyelid gently stretched and slightly everted; the upper lid being drawn a little upward toward the brow, the lower as much downward toward the zygoma. The point carried home to the sac and touching lightly its nasal side, the lids may be left at liberty, while a half circular motion is performed by the instrument; the surgeon neither suffering the point to recede, or on the other hand, allowing it to become entangled in the membrane."

"The probe now rests in a perpendicular direction upon the eyebrow towards its inner angle, and in this direction it is to be gently depressed until it strikes upon the floor of the nostril, where its presence is readily ascertained by a common probe, passed beneath the inferior turbinated bone. The probe of smallest dimensions is of sufficient firmness to preserve its figure in its passage through the healthy duct, but it is too flexible to oppose any considerable obstruction, without danger of a change of figure; for the stricture of the lacrymal ducts it is of sufficient strength." 374.

By several repetitions of the operation, the passage is generally rendered permanent; but should this not be the case, Mr. T. introduces a style having a small flat head, a little sloped, through the punctum lacrymale into the nose, and leaves it for twenty-four hours in the duct. If worn longer, it ulcerates the orifice. A day or two should be suffered to elapse before the style is again introduced, and it should then be passed through the other lacrymal duct. The introduction of tepid water should be made on the intermediate days, by means of Anel's syringe.

We have now, we hope, exhibited as full and comprehensive a view of Mr. Travers's work, as could well be effected within the limits which the article has occupied. The apparently continuous chain of the narrative, may induce a supposition that little art or labour was necessary in the construction of the analysis. Let those who think so, try to imitate it, and they will then feel the difficulty. Mr. Travers's work is so pregnant with sterling information, that we often found it a hard task to compress valuable matter within less bounds than those which it occupied in the original—a rare circumstance in modern publications!

In every part of Mr. Travers's work, there is such forcible indication of good sense and sound judgment, that our assent to his propositions is almost irresistibly secured—and that, apparently without the least effort on his part. Such powers are the lot of few; and we sincerely wish that the excellent author may long enjoy them, for the good of humanity, and the honour of the profession.

The plates are executed in the very best style.

